# Attitudes toward population screening among people living with fragile X syndrome in the UK: ‘I wouldn’t wish him away, I’d just wish his fragile X syndrome away’

**DOI:** 10.1002/jgc4.1355

**Published:** 2020-11-12

**Authors:** Felicity K. Boardman

**Affiliations:** ^1^ Division of Health Sciences Warwick Medical School Coventry UK

**Keywords:** attitudes, expressivist objection, fragile X syndrome, identity, population screening, stigma

## Abstract

In an age of expanded genetic screening, fragile X syndrome is increasingly considered a candidate condition, given its prevalence, the absence of curative interventions, and its impact on families. However, relatively little research has explored the views of families and people living with fragile X syndrome toward population screening. This study reports on in‐depth interviews with 19 participants: 3 with people diagnosed with a fragile X condition (fragile X syndrome = 2, FXTAS = 1) and 16 people with fragile X syndrome in their family (11 parents, 2 grandparents, 1 spouse, 1 sibling, and 1 aunt) living in the UK. This study reveals the complexity of attitudes within this group and the existence of genuine ambivalence toward different population screening programs. While the overwhelming majority believed that preconception and newborn genetic screening should be made available to the general public, the notion of prenatal screening was far more controversial, with only five participants expressing support for such a program. Expressivist concerns were highlighted equally both by those who supported prenatal screening, as by also those who did not. Participants who supported prenatal screening drew clear distinctions between people with fragile X syndrome and the condition itself, in order to neutralize expressivist concerns and existential threat. However, for others, this division was challenging to maintain. Impairment effects associated with fragile X syndrome, more specifically, its implications for behavior, intellect, and personality, made it harder for some participants to conceptually separate the person from their condition. This study concludes that screening remains a complex issue for families living with genetic conditions and that expressivist concerns affect, and are managed by, families living with different types of disability in contrasting ways. Screening for conditions that affect personality, behavior, and intellect produces unique iterations of expressivism, identity, and stigmatization that families produce specific, and creative, strategies to navigate.

**What is known on this topic**

People with genetic conditions should be considered in assessments of population genetic screening, yet are not always consulted. What research has been done has shown support for screening, although conflicts emerge around the ‘expressivist objection’ and the disvaluing of family members through screening support.

**What this paper adds to the topic**

Families living with fragile X syndrome generally support population screening, but with some ambivalence. Intellectual disability produces unique iterations of the dilemmas that face all families living with genetic disease considering population screening, and families develop creative strategies to navigate them.

## 1 INTRODUCTION

Fragile X syndrome is the leading cause of inherited intellectual disability worldwide (Johansen Tab er et al., 2019). An estimated 1 in 291 women in western countries carry the *FMR1* premutation that causes fragile X syndrome (Hunter et al., 2014). Given the significant impact fragile X syndrome has on families, together with the lack of curative treatment, the *FMR1* premutation is considered a prime candidate for population‐based screening programs (preconception/prenatal/newborn) internationally (Hill et al., 2010).

Rapid developments in the world of genomic medicine over the past ten years, including the integration of whole‐genome sequencing into state funded/subsidized healthcare systems, such as the NHS (National Health Service), now mean that expanded genomic screening programs at population level are—at least technologically—feasible. Australia and the Netherlands, for example, have introduced pilots of large‐scale pre‐conceptual genetic carrier screening programs in recent years (Mackenzie’s Mission, 2020; University Medical Centre Groningen, 2016), and such screening programs appear set to proliferate on a global scale.

While expanded genetic screening of the general public is premised on a desire to extend reproductive autonomy and the (currently limited) range of reproductive options available to carrier couples/individuals, decisions around which conditions should be included on carrier panels, and the point in the reproductive pathway such screening should be delivered (e.g., preconception, prenatal), are controversial (Kirk et al., 2020).

Critics of expanded screening programs have highlighted that there may be costs of genomic screening programs to families currently living with genetic conditions. This may be in the form of decreased availability of resources, for example, research into treatments, or social consequences, such as stigma (Kellogg et al., 2014). The transformation of a rare genetic condition into a ‘screened‐for’ condition has also been demonstrated to change its public profile, introducing new ideas about risk to prospective parents, and accountability to those with already affected children. There is evidence, for example, of the felt need among parents of children with Down syndrome, to ‘justify’ their child's existence in light of universal antenatal screening technologies for the condition (Cohen, 2019). Indeed, the potential of these technologies, and the practices that surround them, to ‘express’, or propagate, a negative appraisal of the value of life with disabling traits is at the heart of debates around the so‐called ‘expressivist objection’ (Parens & Asch, 1999). The ‘expressivist objection’ was an argument originally developed to highlight the negative value assigned to disability inherent in prenatal testing and selective termination practices (Buchanan, 1996). However, use of the concept has since been extended to highlight examples of ‘expressivism’ as they relate to other forms of genetic technologies. Indeed, in recent years, accumulating evidence suggests that the concept is relevant to a wide range of current (McNeill et al., 2019), and emerging, reprogenetic practices (Hoffman‐Andrews et al., 2019), and has a substantial impact on the views and decisions of people living with genetic and screened‐for conditions (Boardman, 2014; Boardman & Hale, 2018).

Despite this range of impacts on affected families, studies exploring the social/ethical acceptability of genomic screening have largely focused analytic attention on the views of the general public toward screening (Anido et al., 2007; Fanos et al., 2006; Metcalfe et al., 2017; Ryynänen et al., 1999), with a smaller body of work addressing the views of affected families (Archibald et al., 2013; Bailey et al, 2012; Skinner et al., 2003). While an exploration of public attitudes is clearly essential as the intended recipients of screening, families living with genetic conditions possess direct, and intimate, experience of the condition in question that is of relevance to both the design of screening programs (e.g., determining the conditions are to be included) and their implementation (informing the decision‐making of those screened). The need for consultation with, and an exploration of the views of such families, is therefore paramount in any evaluation of the harms and benefits of future screening programs.

In light of these arguments, this study takes the experiences and views of families and individuals living with fragile X syndrome, as a point of departure through which to explore the acceptability of three different types of population screening program for fragile X syndrome in the UK (preconception, prenatal, and newborn). Using in‐depth qualitative data with people diagnosed with fragile X conditions (*n* = 3) and their family members (*n* = 16), this paper highlights the hopes, concerns, and expectations around screening for those who know fragile X syndrome intimately. It will outline the tensions that emerge within participants’ accounts of screening, particularly as they relate to their lived experiences, personal identity negotiations, expressivism, and stigma, as well as the strategies participants employ to defuse and neutralize them. Through so doing, this paper contributes to an emerging literature exploring the views of people with genetic conditions toward the ever‐expanding world of genomic medicine (Boardman & Hale, 2018), and the role of impairment experiences (i.e., experiences of the impacts of the condition, as separate to the socially created ‘disability’ therein.

### 1.1 Fragile X syndrome

Fragile X syndrome is the leading cause of inherited intellectual disability worldwide and the second most common cause of intellectual disability after Down syndrome (Dykens et al., 2000). Fragile X syndrome results from a CGC repeat in the 5′ untranslated region of the *FMR1* gene and affects approximately 1 in 4,000 males and 1 in 6,000 females.

Individuals with the full mutation (pathogenic variant), that is, those who have over 200 repeats, have fragile X syndrome, which presents as learning difficulties as well as social, behavioral, and emotional difficulties (including ADHD and autism). In addition to these symptoms, fragile X syndrome is sometimes associated with epilepsy as well as mildly dysmorphic facial features.

Males are typically more severely affected than females with fragile X syndrome, with some females exhibiting no clinical symptoms at all. Despite onset of symptoms from approximately 9–12 months of age, the average age at diagnosis is around 36 months, although can be much later (Bailey et al., 2009). Diagnostic delays are often cited as the reason that an estimated 29% of affected families go on to have a second child with fragile X syndrome before the first child is diagnosed, and is a significant argument used in favor of newborn screening (Bailey et al., 2009). While no curative treatment for fragile X syndrome currently exists, various interventions (including behavioral interventions, medication, speech, and occupational therapies, as well as family support) are available to help manage the condition (Hagerman et al., 2009).

### 1.2 Inheritance of fragile X syndrome

Fragile X syndrome is inherited in an X‐linked dominant pattern, as the mutation is located on the X chromosome. Both males and females can be carriers of the fragile X premutation (between 55 and 200 repeats); however, only females can transmit fragile X syndrome to their offspring. Male carriers of the fragile X premutation do not transmit fragile X syndrome; however, each of their daughters will receive the fragile X premutation (and as such may have children with fragile X syndrome themselves).

Unlike most other genetic conditions whereby carrier status is a relatively benign condition, the fragile X premutation is associated with two distinct conditions. In women, the fragile X premutation can cause fragile X‐associated primary ovarian insufficiency (FXPOI), causing irregular menstruation, reduced fertility, and early menopause. Male premutation carriers (and to a lesser extent, females) can experience fragile X‐associated tremor/ataxia (FXTAS) which onsets later in life (typically 50 s/60 s), causing difficulties with motor control, coordination, memory and cognition (Wheeler et al., 2017). It has been estimated that approximately 20%–30% of male carriers of the fragile X premutation develop FXTAS, and approximately 20%–25% of female premutation carriers develop FXPOI (Jacquemont et al., 2004).

### 1.3 Screening acceptability for fragile X syndrome

Screening policies for fragile X syndrome vary internationally. Pilot studies have been conducted with women of reproductive age (Archibald et al., 2009; Metcalfe et al., 2008), pregnant women (Cronister et al., 2005; Hung et al., 2019) and newborn babies (Okoniewski et al., 2019; Saul et al., 2008). However, the lack of curative treatment proves a barrier to screening implementation in many countries. Furthermore, the wide spectrum of severities associated with fragile X syndrome makes the provision of an accurate prognosis following screening extremely challenging for genetic counselors and potentially reduces the utility of the information for parents. Given this complexity, as well as concerns around the screening test itself, no population screening program for fragile X syndrome currently exists in the UK.

A key consideration in the evaluation of screening programs worldwide is the acceptability of that program (Dimmock, 2017). As such, various studies have been conducted exploring attitudes to screening among key stakeholder groups. These have included healthcare professionals (Aacharya & Schindler, 2013), women from the general population (Archibald et al., 2013; Metcalfe et al., 2008), parents of newborn babies (Skinner et al., 2011), premutation carrier women identified through screening (Anido et al., 2007), and parents/relatives of children with fragile X syndrome (Bailey et al, 2012; Skinner et al., 2003).

While these studies provide a useful overview of the perceptions of a wide variety of stakeholders, no studies thus far have included the views of people living with fragile X syndrome, FXTAS, or FXPOI themselves.

This study, using in‐depth individual interviews, incorporates the views of people living with fragile X conditions themselves (*n* = 3), as well as their family members (*n* = 16), and explores attitudes toward preconception, prenatal, and newborn screening for fragile X syndrome.

## 2 MATERIALS AND METHODS

The data for this study were gathered as part of a larger mixed methods study exploring attitudes to genetic screening among families living with a range of genetic conditions. All data were gathered February 2017–March 2018.

### 2.1 Participants

Interview participants for this study were recruited through three separate calls placed in the Fragile X Society UK e‐newsletter at four monthly intervals. The call was also posted on the ‘research’ section of their website. In addition, families who had a registered interest in participating in fragile X‐related research through the Patrick Wilde Centre (a UK research center for fragile X conditions) were contacted via email about the research. Participants were eligible for interview if they were aged over 18, living in the UK, were English speaking and either had a fragile X condition themselves, or had a family member with a fragile X condition (including FXTAS and FXPOI). The definition of family member was kept deliberately broad to include extended family members as well as non‐biological family members such as step and adopted relatives.

The advertisements led to responses from 20 people, and 17 interviews were successfully completed with 19 different participants (two joint interviews were conducted, one with a mother and her son with fragile X syndrome, and another with a couple—Table 1). Two people who initially indicated their willingness to participate failed to respond to attempts to arrange an interview, and one person was excluded as they did not meet the eligibility criteria. Given the relatively low numbers of people with fragile X syndrome in the sample (*n* = 3), snowball sampling was attempted through already recruited relatives; however, this strategy was ultimately unsuccessful as most thought an interview would be too taxing for their family member.

**TABLE 1:** Participant pseudonym, gender, age, relationship to fragile X syndrome, and attitudes toward screening

No.	Name	Gender	Age	Relationship to fragile X	Wider family links to fragile X	Preconception screening	Prenatal screening	Newborn Screening
1	Malcolm	M	56	Parent of 23‐year‐old son with FXS and unaffected daughter (donor conception)	None known	✓✓	✓✓	✓✓
2	Judy	F	51	Parent of 23‐year‐old son with FXS and unaffected daughter (donor conception). Wife of Malcolm	Cousin has FXS. Father has premutation	✓	✓✓	XX
3	Mary	F	75	Spouse has FXTAS	Brother has FXS. Step‐granddaughter with FXS	✓✓	XX	✓✓
4	Sue	F	74	Has FXTAS	Sister has FXTAS. Has two grandsons with FXS	✓✓	X	✓✓
5	Ali	F	48	Premutation carrier, 2 unaffected daughters	One nephew and two nieces with FXS	✓	XX	✓
6	Elisabete	F	31	Mother of 3‐year‐old boy with FXS and unaffected daughter (prenatal testing)	None known	✓	✓✓	XX
7	Emma	F	34	Mother of 14‐year‐old boy with FXS and unaffected daughter (PGD)	Sister premutation carrier	✓✓	✓✓	XX
8	Luca	M	17	Has FXS	Grandfather has FXTAS	✓✓	XX	✓✓
9	Chiara	F	43	Mother of Luca (with FXS). Also has unaffected son (no prenatal testing)	Father has FXTAS. First cousin once removed has FXS	✓	XX	✓✓
10	Joanne	F	47	Mother of two daughters with FXS (15, 12)	Sister has son with FXS	✓✓	XX	✓
11	Valerie	F	66	Mother of son (33) and daughter (29) both with FXS (no prenatal testing)	Cousin with FXTAS and daughter with FXS	✓✓	XX	✓
12	Jane	F	58	Mother of son (25) and daughter (23) both with FXS. Also unaffected son (20) and daughter (19)(no prenatal testing)	Grandson (son of daughter, 23, with FXS) has FXS. Sister has daughter with FXS. First cousin once removed has son with FXS	✓	X	✓✓
13	Sally‐Anne	F	47	Mother of two daughters (15 and 11) with FXS. (no prenatal testing)	Father has FXTAS. Cousin has son with FXS	✓✓	XX	✓✓
14	Zoe	F	40	Premutation carrier with carrier son (16) and unaffected daughter (12)	Two brothers with FXS	✓	XX	✓✓
15	Holly	F	26	Has FXS	Sister has FXS. Two male cousins have FXS	✓✓	—	✓✓
16	Rachel	F	38	Mother of boy (10) with FXS and unaffected boy (2) (no prenatal testing)	Father had FXTAS (deceased). Aunt and uncle both premutation carriers. Six cousins all premutation carriers. Two cousins have sons with FXS and a third cousin currently pregnant with a boy with FXS	✓	XX	✓
17	Tony	M	65	Grandparent of boy (7) with FXS	None known	✓✓	—	✓✓
18	Moira	F	60	Grandparent of boy (10) with FXS	Both daughters’ premutation carriers	✓	XX	✓
19	Kathryn	F	37	Mother of son (4) with FXS (undergoing PGD)	Sister has daughter with FXS. Cousin has son with FXS	✓✓	✓✓	✓✓

Note

✓✓, support for screening; ✓, ambivalent support for screening; XX, non‐support for screening; X, ambivalent non‐support for screening; —, neutral/unable to take a view.

Abbreviations: FXS, Fragile X syndrome; FXTAS, Fragile X‐associated tremor/ataxia syndrome; PGD, Pre‐implantation Genetic Diagnosis.

### 2.2 Interviews

The interview schedule was developed around one previously implemented for families living with genetic conditions considering screening (Boardman et al., 2019, 2020; Boardman & Hale, 2018), and adapted for this population. The questions were passed through an advisory group made up of four fragile X society staff members (including a clinician), and was piloted with a volunteer parent.

Seventeen interviews were successfully completed using a range of different techniques to suit participant needs and preferences. These included face‐to‐face interviewing (*n* = 3), telephone interviewing (*n* = 13), and email interviewing (*n* = 1). Email interviewing, while lacking the visual and audio cues and sense of rapport that can be developed through face‐to‐face or telephone interviewing, enabled the inclusion of a person with fragile X syndrome (Holly), whose social anxiety would have otherwise precluded her participation. Interviews were designed to explore experiences of life with fragile X conditions within families, views and experiences of reproduction and genetic risk and participants were also directly asked to reflect on their perceptions of population screening. As fragile X syndrome is associated with learning difficulties and social anxieties, participation in the research for affected individuals was supported through inclusive interview techniques. These ranged from adapting the method of interview to allow for digital interviewing, speaking to family members before the interview to gauge the participant's communication and comprehension needs and adapting the interview questions in line with these (Barter et al., 2017). For one participant with fragile X syndrome, the interview was conducted with a caregiver present who was able to translate the research questions into everyday scenarios that the participant could more easily relate to, for example, by referring to genetic testing that had already occurred within their own family. These approaches enabled two people with fragile X syndrome (a 17‐year‐old male and a 26‐year‐old female) to contribute their perspectives to the research.

While participants were recruited through the fragile X society, the interviews were all conducted by a white female qualitative researcher, with whom the participants were previously unfamiliar. This unfamiliarity may have prevented the recruitment of further participants with fragile X syndrome (given the condition's association with social anxiety) than if the interviews had been conducted by a familiar support group staff member. However, the separation of the research project from the group may have also afforded the participants greater anonymity, and sense of accountability to the group.

Given the method of recruitment and the possibility of participants being known to one another, particular care was taken to protect the anonymity of those who were involved. After the interviews had been transcribed verbatim, all identifying information was removed from the transcripts and the participants were assigned pseudonyms. Where segments of participants’ stories could not easily be anonymized (e.g., because they were particularly well known within the group), they were either omitted, or ‘participant disguising’ techniques (Weiss, 1994) (e.g., the altering of ages and/or genders) were employed where this would not substantially distort the content, and the emerging themes, of their story. All participants were provided with a copy of their de‐identified transcript for checking, although in reality, only three took up this opportunity.

### 2.3 Data analysis

The data were analyzed using Nvivo 11 software, and a constructivist approach to grounded data analysis was used. This process was inductive, allowing the themes to emerge directly from the research data, although unlike traditional grounded theory analysis, the literature was consulted throughout the analysis process to facilitate theme refinement. After initial ‘open coding’, higher level hierarchical coding was undertaken. A repeated process of coding, refinement of concepts (through data interpretation and insights from the surrounding literature), and re‐coding was completed over a period of three months until data saturation had occurred (i.e., no new concepts were emerging and all of the data were included within the final coding framework) (Glaser & Strauss, 1967).

Upon completion of coding, participant transcripts were assigned quantitative categories according to their support, or non‐support, for the three screening programs (Table 1). Initially, three categories were used to categorize participants (support/non‐support/ambivalent); however, this was eventually expanded to five categories to capture the complexity of attitudes across the dataset. The final categories were support, non‐support, ambivalent support, ambivalent non‐support, and neutral/unable to answer. The categories of ambivalent support and non‐support were developed to capture the views of people who took a view on a screening program (support or non‐support), but who also held considerable reservations, or contradictory feelings about the program. These categories enable a broad overview of the level of support, and its dispersion across the dataset.

This paper presents the core themes derived from the final coding framework of the qualitative analysis along with the quantitative categories. The data from all participants contributed to this final coding framework, irrespective of whether their excerpts are included in this paper. Excerpts have been selected that particularly clearly and eloquently represent the data coded to that theme.

## 3 RESULTS

This section is organized around clusters of core themes emerging from the analysis as they related to the three different screening programs under study.

### 3.1 Newborn screening: avoiding the odyssey or ‘making a well‐child sick’?

Life with fragile X syndrome was almost universally described by participants as a challenging experience, that typically involved a protracted and difficult journey to diagnosis. For the 11 parents of children with fragile X syndrome, the age at diagnosis ranged from 2 to 8 years, averaging 4.5 years. This delay meant that parents were living with the effects of fragile X syndrome, without professional support, for several years. Six of these 11 parents went on to have further children during pre‐diagnosis phase, with four (Joanne, Valerie, Jane, and Sally‐Anne) having further children with fragile X syndrome.

For the majority of the parents (10/11), the long delay to diagnosis meant that it was experienced largely as a relief, and marked the end of the diagnostic odyssey that had plagued their early years with their child. Chiara, aged 43, recalled the diagnosis of her (now) 17‐year‐old son, Luca, with fragile X syndrome (at age six) in the following way:

…that was the hardest time [pre‐diagnosis] for us. My husband and I were constantly blaming each other. We each thought the other wasn't doing enough for him… and the [extended] family back at home [in Italy] didn't believe it, they kept saying he'd grow out of it. We kept getting fobbed off by the paediatrician. So when Luca was finally diagnosed, it was this huge relief. It was saying that yes, we were dealing with something that was very hard, and no, I wasn't imagining it, you know? I had a name for it and could reach out for help and…. that was very healing for us. (Chiara, supporter of newborn screening, mother of son with FXS)

For Chiara, the diagnosis of her son was a validating experience, absolving her and her husband's guilt for having ‘caused’ Luca's apparent difficulties, and was a key reason she gave for supporting newborn screening. Having known earlier, Chiara believed, would have prevented the strained interfamilial relationships that the lack of a clear diagnosis created, and prevented the feelings of isolation that had overshadowed her early parenting experiences.

While ten other participants supported Chiara's view that newborn screening was an important source of information, support, and validation, other participants were more ambivalent in their support (*n* = 5). Three participants did not support newborn screening at all.

Judy, for example, was the 51‐year‐old mother of David (now 23) who was diagnosed with fragile X syndrome at age seven. Judy described David's diagnosis as a ‘traumatic’ event, as it was the point that she realized the permanence of David's difficulties. She described the diagnosis as having a catastrophic impact on her imagined future with her son, triggering a form of grief. Drawing on these experiences, Judy described her thoughts on newborn screening in the following way:

I think you'd be taking something away from them [parent and newborn], actually if you pick it up at birth. You're taking away the normality, when their baby's still absolutely fine…. like making a ‘well‐child’ sick, really. It could really cause all sorts of bonding problems, I think, and that bond can already be a problem for these kids…you know, if I went under a bus tomorrow, David might ask where I was, but then it would be ‘can I have a bag of crisps please?’… I certainly grieved the loss of David, the child I thought I had, because it was then [diagnosis] that I realised this was genetic and permanent…so I think it's better to know when you're pregnant to be honest, so at least you have a choice. If it [screening] isn't giving you options, what's the point? (Judy, non‐supporter of newborn screening, mother of son with FXS)

Judy's account highlights the issues around newborn screening that were both generic—that it does not afford parents any additional reproductive options as compared to other forms of screening—but that were also specific to the unique challenges posed by fragile X syndrome, of which bonding was key. While Chiara experienced her son's diagnosis as validation and as marking the end point of their diagnostic odyssey, Judy perceived a diagnosis in the pre‐symptomatic phase of fragile X syndrome as an extension of the syndrome‐ as making a ‘well‐child sick’.

Seven participants raised the possibility that newborn screening would offer reproductive choice in subsequent pregnancies for new parents. Although, for some, this knowledge was described as a double‐edged sword. Joanne was 47 at her interview, and the mother of two girls, Chloe (15) and Tess (12), both with fragile X syndrome. Chloe had not been diagnosed when Joanne became pregnant with Tess, so she was unaware of the possibility of recurrence. Reflecting on newborn screening, Joanne commented:

…In hindsight now I’m glad I didn't know that Tess [second child] might have it too. We had no idea it could happen again‐ we probably wouldn't have had her. Who knows? But what I do know is that not having Tess would have been a massive mistake, so I have mixed feelings about it, but I can see that it is important for other people to give them options for the next child, so yeah it's probably a good thing on balance. (Joanne, ambivalent supporter of newborn screening, mother of two daughters with FXS)

The idea that newborn screening could have altered the course of their reproductive trajectories and the composition of their families left participants such as Joanne with ambivalent feelings. The rhetoric of information as universally positive and empowering needed to be reconciled alongside the possibility that genetic risk information might have prevented their subsequent child's existence, irrespective of fragile X status.

### 3.2 Preconception screening: information, education, and stigma

Unlike newborn screening, preconception genetic screening garnered support from all participants across the dataset (although eight expressed ambivalent support) and emerged as the most popular screening format. Rather than expanded reproductive autonomy, however, most participants positioned their support for preconception screening in terms of information and education, as Holly, a 26‐year‐old with fragile X syndrome commented:

I would say it's a good way to give people the knowledge they need early on. Lack of awareness is a big problem, so the more people who know the better I would say. If you know early, you have time to find out a bit more about it and do your research and it's not a shock. (Holly, supporter of preconception screening, has FXS)

As well as informing the general public and raising awareness, preconception screening was also seen as a way that carriers could be alerted to the possibility of developing premature ovarian failure—a fact that participants felt was relevant to women's reproductive planning. Ali is a 48‐year‐old carrier with two unaffected children. She discovered her premutation status following the diagnosis of all three of her sister's children with fragile X syndrome. Ali commented:

I think it's quite important that women know early on if they've got it [premutation], because if they want kids and they're one of them that gets infertility, they'll feel they've missed out …but on the other hand, it could also cause a lot of pressure if she hasn't got a partner yet, or a partner is put off. There's quite a stigma to it all, and it's a lot on that woman's shoulders. I got off quite lightly really because I’m a carrier, but I didn't know, and I’ve had two healthy girls. So although I’m glad I didn't have to worry for nothing, I still want both my girls tested when they're old enough‐ for their own health first and foremost. (Ali, ambivalent supporter of preconception screening, carrier)

The potential stigma and distress of discovering carrier status, which Ali perceived could become highly gendered in a preconception screening context, had to be weighed up in Ali's mind against the potential health benefits she perceived for her daughters by identifying their propensity to develop FXPOI or FXTAS later in life. Ali's acknowledgement of relief that she had not known her own status highlights the tensions inherent within this balancing act. Indeed, there was widespread evidence within the dataset of the shame, guilt and stigma that was associated with the transmission of fragile X through families, which participants discussed in the context of preconception screening.

Jane was 58 at the time of her interview and had five people in her family diagnosed with fragile X syndrome, including two of her four children, one grandchild, a niece, and a cousin. Her father, now deceased, had been diagnosed with FXTAS. Jane described fragile X as being somewhat of a ‘shameful secret’ within her extended family, and one tinged with stigma, guilt, and blame. This, in turn, fed into her hopes and expectations of what preconception screening could deliver:

People in my family in past generations were either hidden away and you didn't speak about them, or they were put in institutions, which is awful. But perhaps, you know, screening would open that up a bit, because I feel you owe it to your family members, if you're a carrier, to pass that information on, which doesn't always happen. I don't know why it's such a shameful secret. I know my dad did. It came from him originally, so I think he felt he had cursed this whole family. And I would hope that screening might start those conversations…if everyone is automatically screened. (Jane, supporter of preconception screening, mother of son and daughter with FXS)

For Jane, preconception carrier screening was seen as providing a means to enact one's ‘genetic responsibility’ (Etchegary et al., 2009)—an obligation that she felt existed between biologically related kin, and yet was being mitigated against by stigma, guilt, and blame. The language of ‘curse’ shows how heavily entrenched the negative ideas about transmission were, exacerbated by the dominant inheritance of the condition and the identification of a single ‘original’ carrier within the family. Indeed, there was some evidence across the dataset that older generations, particularly grandparents, felt this more keenly, and took responsibility when the condition cascaded down subsequent generations of their family. By viewing screening as something that would eventually become accepted as routine, or ‘automatic’, Jane hoped that testing for the premutation could become detached from the familial guilt that currently overshadows it.

The stigmatization of fragile X, however, was not contained within affected families. Participants provided numerous examples of stigma that pervaded all aspects of their daily lives. As has been reported with other cases of ‘invisible disability’, such as autism (Gray, 2002), participants felt that their children were being judged as ‘naughty’ (Emma) or ‘out of control’ (Moira) or that they were being judged as ‘bad parents’ (Malcolm) if their child became overwhelmed, behaved in unexpected ways, or had a ‘meltdown’ in public spaces. For some participants (Kathryn, Valerie), anticipation of this stigma regularly prevented them from leaving the house.

For many participants, this awareness of the stigmatization of fragile X fed into their expectations of screening. Five participants reported that while nearly all disability is stigmatized, that conditions involving learning/behavioral difficulties attract particularly heightened forms of stigmatization. Zoe (40) a premutation carrier with two brothers with fragile X syndrome commented:

I think personally think [preconception] screening will be really important to the public and people will want to have it, because look how many have the down syndrome screen. There's just less understanding of mental disability. People think they'll never be able to communicate, they'll always be dependent, they're violent, they'll be a burden, …as soon as they know it causes behavioural problems and things like that then, yes, they'll want to screen for it. People don't want kids with them problems, do they? (Zoe, ambivalent supporter of preconception screening, carrier)

Whereas Jane perceived that stigma could be a barrier to family communication about genetics, for Zoe, stigma was considered an impetus both for the introduction, but also the uptake, of population screening. Intellectual disability emerged as being marked out for very particular forms of stigmatization, separate to, and yet more intense than that assigned to physical disability.

### 3.3 Prenatal screening: autonomy, expressivism, and identity

While preconception and newborn screening garnered broad support from most participants, the notion of prenatal screening was far more divisive due to its association with selective termination. Five participants stated that they would support a prenatal screening program, with the remainder of the participants (12) expressing non‐support, or ambivalent, views. Two stated that they felt too conflicted to express a view at all. All of the participants (including those who supported prenatal screening) grappled with the perceived contradiction between their desire to increase information and reproductive autonomy for the general population with the notion that screening might have prevented their child's existence, and that it's very availability expresses disvalue of that existence. It is notable, however, that screening supporters and non‐supporters resolved these conflicts in contrasting ways.

Kathryn, 37, had a four‐year‐old son, Jake, diagnosed with fragile X syndrome at the time of her interview (alone with two other children diagnosed with the condition in her extended family), and was undergoing her second cycle of PGD. Kathryn explained her supportive views toward prenatal screening, while also acknowledging expressivist concerns:

I think doing it [screening] prenatally is fine as long as….I think full information about the condition in all its forms is really important to make good decisions. As much as I find it upsetting, you have to remember this a disease you're getting rid of, not a person, and it's a disease that can be debilitating for the whole family. This is why we're currently in our second cycle [of PGD] to stop… I mean, of course I wouldn't wish Jake away, I’d just…I’d wish his fragile X away. (Kathryn, ambivalent supporter of prenatal screening, mother of son with FXS)

Like the other four prenatal screening supporters, Kathryn indicated the importance of high quality and unbiased information about all forms of fragile X syndrome as a vital resource to support any prenatal decision about the condition. While some participants who did not support prenatal screening queried whether the level of information required for ‘good’ decision‐making was ever a realistic goal for the general population (Emma), screening supporters generally reported that a right to reproductive autonomy overrode concerns regarding how fully informed those decisions ultimately were. For Kathryn, the mother of a child with fragile X syndrome, expressivist concerns associated with prenatal screening emerged as salient within her account. In order to manage them, Kathryn drew a clear ideological distinction between Jake himself, and fragile X syndrome. By so doing, Kathryn was able to clarify that the true target of selective reproductive practices was a ‘debilitating disease’ not a person, enabling her to deflect any critical appraisal of fragile X syndrome away from her son's life and consequently reconcile her view of screening with her support and love for her son.

However, for other participants, the distinction that Kathryn relied on—between a person and their condition—did not hold, particularly in the context of the unique impairment effects that were associated with fragile X syndrome. Rachel (38), the mother of a 10‐year‐old son, James, with fragile X syndrome and a second (unaffected) son (born without prenatal testing), felt that prenatal screening had eugenic undertones given the inseparability of people from their condition:

I think prenatal screening would ultimately be used as a way to get rid of people with fragile X syndrome, and I find that very hard to swallow. These kids have so much to offer and have as much right to be here as the next person. You know, with James, fragile X is part of his personality, his temperament, his interests, his behaviour. You can't think of it as something you can just get rid of and then you have a healthier version of your child… If I were to have James without it, would I even know who he was? It makes him who he is. (Rachel, non‐supporter of prenatal screening, mother of son with FXS)

Unlike Kathryn, who conceptually separated out personhood from fragile X syndrome, Rachel viewed fragile X syndrome as so much a part of her son's existence that it could not be extracted. Whereas Kathryn had presented fragile X syndrome as a debilitating ‘disease’, Rachel conversely associated the fragile X mutation with positive characteristics that she perceived to have been passed down through her family. Reflecting on her father's experience of FXTAS, she commented:

My dad was a really lovely person, no side to him, just so giving and loving, you know, and you see that in people with fragile X… Hopefully I'm a little bit like that too, as a carrier, and if I am, then that's the bit about me that I like. I think if you start trying to change things, you start screening for stuff, yes you might get rid of some of the problems, but you also lose the good bits as well. Very rarely is something 100% completely bad. (Rachel, non‐supporter of prenatal screening, mother of son with FXS)

While the genetic origin of familial diseases such as fragile X conditions has been demonstrated to disrupt kinship relationships within families through the existence of blame, stigma, and guilt (e.g., Jane), Rachel's account conversely highlights how it could also be interpreted as affirming; confirming familial ties and integrating the genetic mutation into a family legacy that consciously upheld the value of affected family members, while also deflecting the expressivist threats posed by prenatal screening.

## 4 DISCUSSION

Overall, this paper presents the multi‐faceted, and frequently ambivalent, attitudes of families living with fragile X syndrome toward three different types of population screening program: preconception, prenatal, and newborn. Greatest support was shown overall for preconception screening, followed by newborn screening, whereas prenatal screening was far more divisive, confirming the findings of other studies in this area (Skinner et al., 2003; Bailey et al, 2012). However, the findings also highlight the range, and complexity, of the tensions that underpinned these participants’ screening support and non‐support, as well as the ways they negotiated and resolved them. It is these tensions that may be missed by reliance on survey methods alone to explore screening attitudes. Yet, they are key to highlighting the wide range of factors that families living with genetic conditions must navigate when considering population screening for the condition they live with.

The data, for example, starkly illuminate the role of impairment, and impairment effects, in shaping the nature, and content, of the nuances around screening that emerged through participants’ responses to all three screening programs. Previous research on screening attitudes among families and individuals living with different types of genetic condition has revealed some common themes in their responses to screening. Research exploring the views of people and families living with conditions as diverse as spinal muscular atrophy (Boardman et al., 2017) and inherited retinol conditions (Hoffman‐Andrews et al., 2019), for example, demonstrates that despite the wide spectrum of impairment effects associated with these conditions, common themes can be identified. These themes include a querying of presumed low quality of life with genetic disease (Boardman & Hale, 2018; Hoffman‐Andrews et al., 2019), the role of social factors—notably stigma and environmental barriers—in shaping life experiences (Boardman et al., 2020), the dire need for greater public awareness of the condition (Boardman & Hale, 2018), and the key role of expressivist concerns in framing screening responses (Hoffman‐Andrews et al., 2019). Many of these core themes were also echoed in the accounts of families living with fragile X conditions. However, this present study adds to this literature by also illuminating the unique iterations of stigma, identity politics, and expressivism that emerge within the screening attitudes of families living with impairments that are associated with cognitive and behavioral differences, rather than those which are predominantly or entirely physical in presentation.

Positive attitudes toward newborn screening for fragile X syndrome have previously been identified in the literature (Bailey et al., 2012; Carmichael et al., 1999; Skinner et al., 2003) and were largely confirmed by this study. This support was largely driven by a desire to reduce uncertainty associated with the pre‐diagnostic phase of the condition and, to a lesser extent, inform reproductive planning. However, the data also revealed concerns regarding the impact of an early diagnosis for families. The psychosocial effects of a newborn diagnosis in a pre‐symptomatic infant have been noted in the wider literature on newborn screening, with implications for parental acceptance and adjustment to the condition (Grob, 2008), as well as the loss of enjoyment of the pre‐symptomatic phase (Carmichael et al., 1999)—which may be as long as 12 months for fragile X syndrome. Three participants raised such concerns about newborn screening within this sample. Given the potential impact of fragile X syndrome on the development of affective bonds (described vividly by Judy), the issue of parent/child relationship formation after a newborn diagnosis might be more of a concern to this group than for other rare disease groups for which newborn screening is being considered.

Preconception screening was the program that garnered most support from participants, with the need to increase public information and reproductive autonomy the key drivers behind this support. As has been acknowledged in the literature, however, there are many challenges associated with the implementation of preconception screening; public knowledge of fragile X syndrome remains low (Archibald et al., 2013, 2016), and a lack of a family history with the condition is still a barrier to screening uptake, despite its irrelevance. This study adds that the stigmatization of conditions that involve learning, behavioral, and cognitive differences, over those that are entirely physical, was perceived by participants to be both a key driver toward preconception screening uptake, yet also confirmed and reinforced through that screening. By transforming fragile X syndrome into a ‘screened‐for’ condition, participants raised the possibility that the heightened stigma they perceived to be assigned to cognitive/intellectual disability, would be further underscored and left unchallenged. Indeed, public misunderstanding and ignorance of the impacts of fragile X syndrome punctuated the accounts of all the participants in this study, and underpinned much of their support for preconception screening, in the hope of raising awareness and understanding, as well as dispersing some of the shame and guilt experienced within fragile X affected families, particularly among older generations.

It is perhaps unsurprising—given its association with selective pregnancy termination—that prenatal screening emerged as the most controversial form of screening, and one which provoked the highest degree of ambivalence from participants, confirming the findings of other research (Bailey et al., 2012; Skinner et al., 2003). Despite the high value that participants placed on reproductive autonomy and choice, they nearly all struggled with the notion that prenatal screening could be interpreted as a negative appraisal of the value of people with fragile X syndrome—its expressive potential.

Both participants who supported prenatal screening—as well as those who did not—managed this tension by differentiating the boundaries between personhood, identity, and fragile X syndrome, although they did this in completely different ways. For the five participants who supported prenatal screening, being able to conceptually disentangle people with fragile X syndrome from the pathology that caused it became a strategic device to neutralize the threat that prenatal screening posed to the value of their affected relatives. By viewing screening as targeting, and potentially eradicating, a disease rather than a person, prenatal screening supporters were able to subjugate the distress they felt at supporting a technology that could have, at least in theory, been used to prevent the birth of their own child.

Participants who did *not* support prenatal testing, however, or were ambivalent toward the technology, also drew on discourses of personhood, identity, and genetic conditions to support their position, although they did this in an entirely different way. For these participants, the very nature of fragile X syndrome, more specifically, its impact on personality, learning, and behavior, meant that the separation of the person from the condition was considered an impossibility. For these participants, fragile X was deemed to create particular types of people—‘*the Lucas of this world*’ (Chiara)—who were united by their similarity of traits, not only to other people with fragile X conditions within their own family, but also across other fragile X affected families. As such, it was inconceivable, for these participants, that the condition and the person be considered two separate entities.

These unique forms of biosociality and kinship identification (Featherstone et al., 2006) were interpreted in different ways. For some, they were a source of shame and guilt (Jane), for others, they formed part of a family legacy that also carried positive attributes (Kathryn). Whether they were interpreted positively or negatively however, these characterizations nevertheless reinforced the indivisible nature of the person and the condition, meaning that prenatal screening practices (when used for the purposes of selective termination) were more closely tied to a negative judgment on the value of particular types of people.

Taken together, these findings suggest that the ‘expressivist objection’ to prenatal testing and screening may be perceived and experienced in different ways across disabilities. While it has long been acknowledged that the nature of an impairment (in particular its age of onset and its relative stability over time) has a significant impact on the degree to which disabled people incorporate it into their identity and sense of self (Bogart et al., 2017; Hoffman‐Andrews et al., 2019), this study suggests that other impairment variables, specifically whether the impairment is physical or cognitive in nature, may also impact the degree to which expressivist concerns around genetic screening are experienced, interpreted, and responded to. While previous research has demonstrated that the higher the degree of perceived integration between self and impairment, the greater the significance of expressivist concerns within reproductive views and decisions (Boardman & Hale, 2018), this study adds that for conditions that affect behavior and personality, the blending of personhood with condition results in very particular experiences with expressivist concerns and, consequently, strategies of deflection.

### 4.1 Conclusions and Practice Implications

Overall, therefore, this study demonstrates the complexity of the views of families and adults living with fragile X syndrome toward population screening programs and the significance of impairment effects in this process. This study highlights the need to consider the nature of the impairment in question and its wider social positioning in terms of stigma when interpreting the responses of these families. Conditions that involve learning, behavioral, and personality differences have unique impacts compared to those that are physical, and for participants in this study, resulted in very specific iterations of expressivism, identity, stigma, and reproductive dilemmas, brought to the fore by a discussion of screening. Tapping into this wealth of expertise and insight possessed by families and individuals such as these may be used to better understand, anticipate, and address the increasing complexity of reproductive decision‐making in an age of expansive genomic medicine.

### 4.2 Strengths and Limitations

This study is strengthened by the range of relationships to fragile X conditions within the sample, and the inclusion of people with fragile X syndrome and FXTAS themselves. Moreover, the sample had considerable experience with fragile X conditions to draw on, with 13 of the 19 participants (68%) having two or more affected relatives.

In spite of this, however, the final sample, and methods of recruitment, pose certain limitations.

In the first instance, the sample size was relatively small overall, which limits the transferability of the findings. Indeed, perspectives on screening among families living with fragile X conditions are multiple and complex, and this study represents a small sample of views. However, by focusing on a small sample of participants, this analysis was able to achieve depth of analysis that would have been precluded by a larger sample size.

Secondly, despite concerted efforts to include people living with fragile X conditions, the number who participated in the study was also small overall (*n* = 3). Two participants had fragile X syndrome and one had FXTAS. Though snowball sampling was attempted to increase numbers, this was ultimately unsuccessful. Given the demanding nature of the research topic, however, both in terms of intellectual and emotional complexity, the successful inclusion of two people living with fragile X syndrome could ultimately be considered a strength of the project.

Thirdly, parents of children newly diagnosed with fragile X syndrome were notably under‐represented in the sample; 7/11 parents had children aged 15 or over at the time of interview. This may have influenced attitudes to newborn screening as their children were diagnosed before 2007, the point at which genetic testing became more accessible (Gabis et al., 2018). Indeed, the average time to diagnosis for this sample was 4.5 years, longer than the average of 3 years reported in the literature (Bailey et al., 2009).

Finally, this study is further limited by its reliance on a support group and a UK research center mailing list as the means of recruitment, meaning that there was a degree of self‐selection to the sample.

Despite these limitations, however, the final sample produced a rich and diverse dataset, highlighting a range of perspectives and experiences with fragile X syndrome and *FMR1*‐associated conditions.

### AUTHOR CONTRIBUTIONS

As sole author, FB undertook all work associated with this manuscript from design and conception of the study, oversight of data collection, data analysis, writing, and editing. FB accepts sole responsibility for data integrity.

### COMPLIANCE WITH ETHICAL STANDARDS

#### CONFLICT OF INTEREST

FB confirms she has no conflicts of interests to declare.

### HUMAN STUDIES AND INFORMED CONSENT

Ethical approval for the study was sought from the Biomedical and Scientific Research Ethics Committee (University of Warwick) and was granted in November 2016. This study was conducted in compliance with the University of Warwick's research ethics guidelines, and informed consent was sought, and documented, from all participants before interview. No identifiable data have been included in this manuscript.

### ANIMAL STUDIES

No non‐human animal studies were carried out by the authors for this article.

### DATA SHARING AND DATA ACCESSIBILITY

The data presented in this study are not currently deposited in a data repository, but will be upon completion of the research. Until then, the data are available from the corresponding author upon reasonable request.
